# Characterizing the kinetics of presepsin and associated inflammatory biomarkers in human endotoxemia

**DOI:** 10.1007/s00011-022-01610-1

**Published:** 2022-07-21

**Authors:** Linda B. S. Aulin, Anne Kleijburg, Matthijs Moerland, J. G. Coen van Hasselt

**Affiliations:** 1 Leiden University, Leiden, The Netherlands; 2 Centre for Human Drug Research, Leiden, The Netherlands; 3 Leiden University Medical Center, Leiden, The Netherlands

**Keywords:** LPS, Host response, Biomarker, Presepsin, Kinetics

## Abstract

In this study, we describe the kinetics of a new potential inflammatory biomarker, presepsin, together with a panel of well-established biomarkers in a human endotoxemia study. We evaluated biomarker correlations and identified combinations that could hold valuable insights regarding the state of infection.

## Introduction

Severe bacterial infections and sepsis are characterized by a systemic immune response. Inflammation-associated proteins can hold potential as treatment efficacy biomarkers. Such biomarkers can be utilized to monitor antibiotic therapy and inform treatment optimization, aiming to improve outcomes in patients [1]. It is essential that treatment efficacy biomarkers have a short induction time, a relatively rapid half-life, and closely follow the course of infection to reflect the current state of infection.

The current biomarkers of infection, such as leukocyte counts and C-reactive protein (CRP), are either non-specific or suffer from a delayed onset of production and slow half-life. Identifying novel biomarkers with more favorable properties could improve the utility of biomarker-guided treatments in severe infections and sepsis. Additionally, using a combination of different biomarkers has been shown to improve predictive performance [2], suggesting the importance of understanding correlation in biomarker kinetics. Presepsin is emerging as a potential biomarker to inform treatment of infections and sepsis, and is associate with clinical disease severity [2, 3]. However, the kinetics of presepsin, and how it relates to other established biomarkers, is poorly understood.

The clinical utility of treatment response biomarkers for infection and sepsis is currently hampered by poor characterization of their kinetics. Characterizing biomarker kinetics in severely ill patient is challenging due to the heterogeneity in underlying infection or disease state. Experimental endotoxemia in healthy volunteers resembles some features of the inflammatory response during infection and sepsis. In this model, a systemic toll-like receptor 4 (TLR4)-mediated inflammatory response is induced by administering lipopolysaccharide (LPS). Although LPS challenge studies are considered a poor model of clinical sepsis, these studies may help to characterize specific components of the inflammatory responses, which play an important role in sepsis. In the current study, we evaluated presepsin response in a human LPS challenge model, related the response to more standard inflammatory biomarkers, and characterized the kinetics of the response.

## Materials and methods

This is a sub-study of a larger previously published study [4]. This sub-study included ten healthy male volunteers who received a single dose of 1 or 2 ng/kg bodyweight of LPS. Blood samples were collected pre-LPS exposure up to 24 h post LPS administration. Further study details can be found in the primary publication [4].

We quantified plasma concentration–time profiles of presepsin by ELISA (Abbexa, abx76557, limit of quantification (LOQ) = 31.25 pg/mL). Inflammatory cytokines (IL-1β, IL-1Ra, IL-6, IL-8, and TNF-α) and CRP were analyzed using electro-chemiluminescence assays, as previously described [4].

We calculated the total area under concentration–time curve (AUC) post LPS administration for each individual volunteer and biomarker, using the trapezoidal method with observations below the LOQ imputed by LOQ/2. We compared AUC between the dose groups (Wilcoxon rank-sum test, *α* = 0.05), and calculated the Pearson’s correlation (*r*) between the different biomarkers and applied hieratical clustering.

## Results

**Fig. 1:**
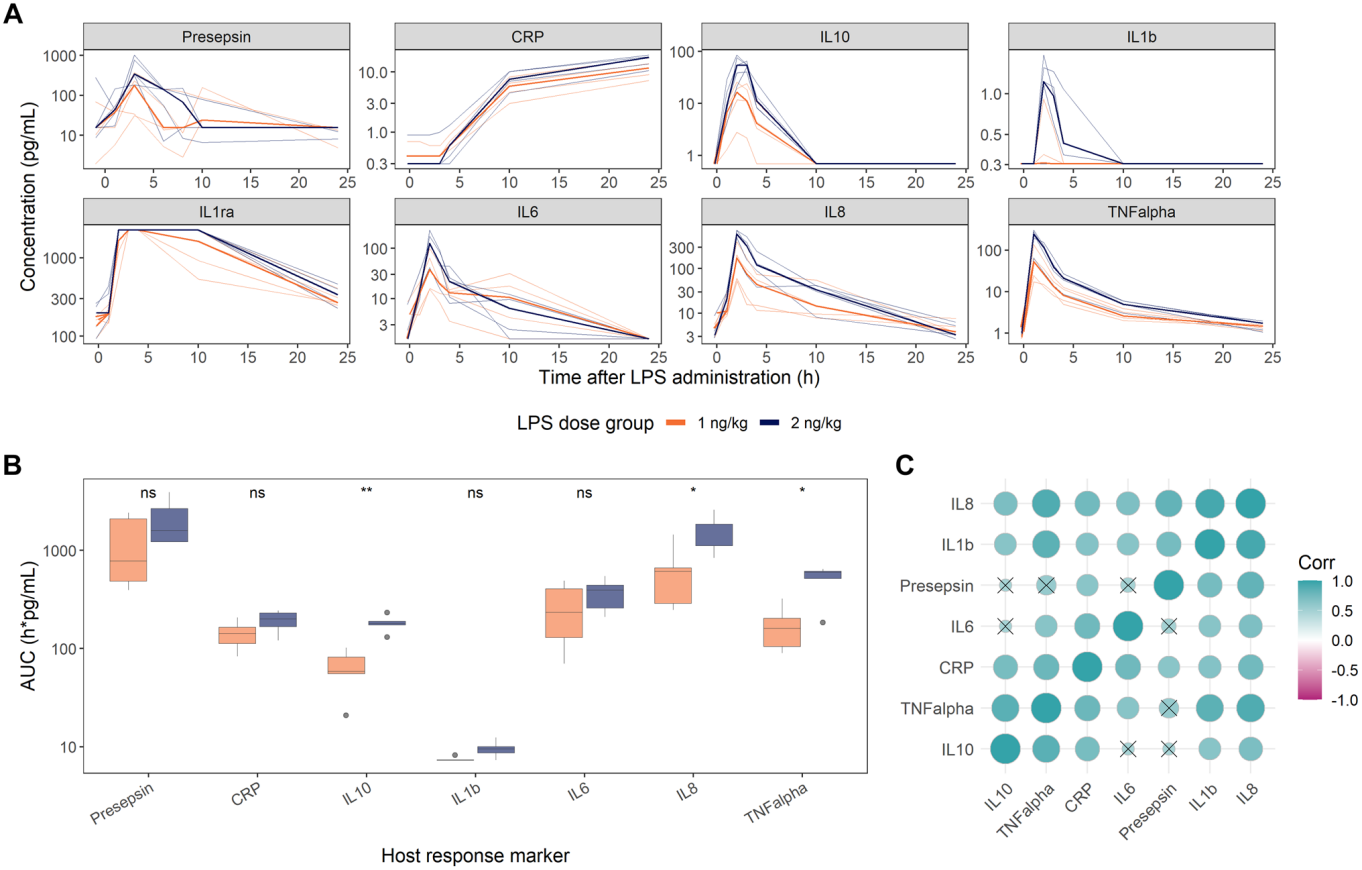
Lipopolysaccharide (LPS)-induced host response in healthy volunteers (*n* = 10). **A** concentration–time profiles of median (solid line) and individual (shaded lines) concentration of host response markers. **B** 24-h post-LPS area under the curve (AUC) per host response marker, with dose group comparison (the Wilcoxon rank-sum test), ns: *p* > 0.05, *: *p* ≤ 0.05, **: *p* ≤ 0.01 **C.** Hierarchically clustered Pearson’s correlation (Corr) matrix of host response marker AUCs, X: *p* > 0.05

An increase for all measured inflammatory biomarkers was observed (Fig. 1A), with an observed maximum response within 10 h post LPS administration except for CRP. For IL-1ra, its peak exceeded the upper LOQ of 2250 pg/mL and was therefore excluded from the AUC calculations.

We identified significant effect of LPS dose and mean biomarker AUC for TNF-α, IL-10, and IL-8 (Fig. 1B), where the relative difference in AUC between the dose groups was 290%, 285%, and 252%, respectively. A positive correlation between individual-level AUC was observed for all biomarkers (Fig. 1C). The AUC of TNF-α was highly and significantly (p < 0.05) correlated to both IL-1β, IL-8, and IL-10 (*r* ≥ 0.85). These findings align with previous in vitro studies where IL-1β and TNF-α induce each other’s expression [5, 6] and TNF-α enhances IL-8 expression, while anti-TNF drug has been shown to supress LPS-induced IL-8 secretion [7]. The strong correlation between IL-1β and IL-8 (*r* = 0.94) could be a result of TNF-α-related induction, but could also be affected by direct interaction between the two inflammatory markers. Presepsin was most strongly correlated with IL-1β (*r* = 0.73) and IL-8 (*r* = 0.82), and all three markers clustered in the correlation analysis. Due to the strong correlation, the clinical value of these biomarkers could potentially be increased when used in combination.

## Discussion

In this study, we describe the kinetic presepsin, together with a panel of well-established biomarkers. To our knowledge, this is the first study that describes human presepsin kinetics in healthy volunteers after LPS administration. The study adds value as simultaneously measuring levels of a large panel of host response markers in the same individuals allows for the evaluation of individual-level biomarker correlations. Such correlations can aid in the understanding of the complex interplay between biomarkers, as demonstrated by the identification of known interactions between TNF-α, IL-1β, and IL-8.

Our study builds toward an increased understanding of presepsin kinetics in reposes to LPS and how it relates to established inflammatory biomarkers. Although our study only covers a part of the complex picture of immune response to infection by design, it provide an important piece of the puzzle and endorses presepsin as treatment response biomarker candidate. The rapid pronounced induction and short half-life of presepsin support further studies of presepsin as biomarker for systemic inflammation. Previous studies have reported higher presepsin levels in patients with infection compared to non-infected patients [3], thus indicating specificity for infections. Presepsin specificity gives it an advantage as a potential treatment response biomarker over more general biomarkers of inflammation, such as CRP. Although no longitudinal leucocyte data were collected in this study, we acknowledge the value of comparing prespsin kinetics with this standard of care biomarker.

In conclusion, our findings characterizing the kinetics of inflammatory markers may constitute a step toward understanding parts of the TLR4-mediated immune response during an infection. Such understanding can contribute toward the efforts of interpretation of clinical inflammatory biomarkers, which in future could play a role in informing treatment optimization and individualization for patient with severe infections or sepsis.
